# A Simplified, Specific HPLC Method of Assaying Thiamine and Riboflavin in Mushrooms

**DOI:** 10.1155/2019/8716986

**Published:** 2019-02-03

**Authors:** Mohammad F. Hossain, Mamoon Rashid, Rajjit Sidhu, Randy Mullins, Susan L. Mayhew

**Affiliations:** Appalachian College of Pharmacy, Oakwood, VA 24631, USA

## Abstract

Mushrooms have been used as part of the average diet and as a nutraceutical for thousands of years due to their immense health benefits. The purpose of this study was to develop a simple, fast, accurate, specific, reproducible, and robust chromatographic method to identify and quantify two water-soluble vitamins: thiamine (B1) and riboflavin (B2) in mushrooms. The method employed for qualitative and quantitative analysis of these vitamins was Reversed Phase-High Performance Liquid Chromatography (RP-HPLC) equipped with Ultraviolet–Visible (UV-Vis) Detector. The extraction process involved acid hydrolysis followed by enzymatic dephosphorylation with takadiastase enzyme. Chromatographic separation was achieved with a Shimadzu prominence HPLC system using isocratic elution mode on a Waters Xterra® MS C-18 column (4.6mm × 150mm, 5 *μ*m) integrated with a XBridge® BEH C-18 Guard column (2.1mm × 5 mm, 5 *μ*m). The mobile phase of this study consisted of buffer and methanol in the ratio of 80:20, where the buffer contained sodium-1-hexanesulfonate, glacial acetic acid, methanol, and pH adjusted to 3.0 with diethylamine. Vitamins were detected simultaneously at their lambda max wavelengths B1: 245nm and B2: 268nm using dual-wavelength UV detection technique to get their highest response. The proposed method was found to be specific, linear R>1.0, accurate, precise (% recovery ± SD; B1:104.45±4.5 and B2: 104.88±2.04), sensitive, (limit of detection for B1 and B2 was 0.043 and 0.029 *μ*g/mL, respectively), and robust for mushrooms analysis. No coeluting peaks were observed at the retention time of the vitamins and all the peaks were spectrally homogenous. The standard and sample solutions were found to remain stable at cold temperature for 72 hours. In summary, our data suggest that the proposed method could be used in food industries to monitor the product quality during routine quality control purposes.

## 1. Introduction

Mushrooms have been used as staple ingredient in diets around the world for their nutritional and medicinal benefits [1]. Recently Western culture has been emulating Eastern culture in their use of mushrooms, due to their use as a health food and for their abundance of vitamins. Oriental culture has historically placed importance on* Lentinus edodes* commonly referred to as the shiitake mushroom. They believe that the mushroom has anticancer, antiviral, immunopotentiating, hypocholesterolaemic, and hepatoprotective properties [2]. Mushrooms are known to contain B vitamins such as thiamine (B1), riboflavin (B2), niacin (B3), pantothenic acid (B5), and folate (B9), all of which are important for proper metabolism and cognitive functioning [3, 4]. Since the early 1990s researchers and consumers alike have recognized the health benefits of mushrooms, and there has been an increasing trend to incorporate mushrooms into the daily diet [1, 5–7] and with the increased consumption of mushrooms especially as part of the western culture, the US has become the second largest producer of mushrooms worldwide [8]. In the US, there is one main species of mushroom that is cultivated, the white button mushroom (Agaricus bisporus), and its two subspecies the Portobello and Crimini variety as found by the USDA (2003) [9]. With consumers becoming more health conscious and looking at the medicinal benefits of mushrooms, corporations have begun to look at extracting vitamins from mushrooms and placing them in capsule form [5, 10]. Hence, the importance of quantifying vitamin content and providing consumers with a product that has undergone quality control testing. Two water soluble vitamins, B1 and B2, are easy to extract and measure form nature products using HPLC [11–18]. This is why these two vitamins were chosen to be the subject of the study.

When food is consumed, it is subjected to two different types of digestion, mechanical and chemical. Mechanical digestion involves the mastication and swallowing of food, whereas the chemical digestion includes the use of acids and enzymes to break down food particles. It is during the acid hydrolysis in the stomach that vitamins are released from the food. Finglas and Faulks (1987) showed that similar to normal digestion, vitamins need to be exposed to hot acid to be converted into their free form [19]. Enzymatic hydrolysis in the form of takadiastase enzyme was used to break the phosphate esters found in proteins. The extraction methodology used in this study was validated and reported in Esteve, Farré et al. 2001, where vitamins B1 and B2 present in mushrooms were identified and quantified by a fluorescence detector [20]. The extraction and chromatographic procedures by fluorescence detection were again verified by Furlani and Godoy in 2008 [21]. Vitamins present in food and drug supplements are detected either by fluorescence detector or by UV detector or by both techniques simultaneously [18, 21, 22]. Officially, the USP/NF has both UV and fluorescence detection methods to determine vitamin content in pharmaceutical dosage forms [11, 13]. HPLC with fluorescence detection has been used widely for the determination of B1 and B2 in foodstuffs; however, fluorescence detector is much less common than UV detector. UV-Vis detector is the most common and widely used detector in research, food, and pharmaceuticals laboratories [23]. Nationally recognized as the “go to” source for drug information, the United States Pharmacopeia/National Formulary contains drug monographs that can provide a starting point for research, as it can provide a background information about the chromatographic conditions for the related compounds, which can be changed to suite the particular needs of the researcher. Of importance is the chromatographic information that it contains, and the analytical methods that can be used to develop analytical methods for the identification and quantification of related or similar compounds in food and pharmaceutical products. Since natural products contain thousands of compounds, specificity of the analytical method is considered to be one of the important parameters. After several unsuccessful attempts using the published chromatographic method, an attempt has been taken in our lab to modify the USP chromatographic method for water-soluble vitamin capsules and oral solution and to develop a simplified, faster, accurate, specific, and highly sensitive method to determine the content of vitamins B1 and B2 present in mushrooms using dual-wavelength UV detection technique [11, 13].

## 2. Materials and Methods

### 2.1. Materials

All solvents used were HPLC grade and analytical reagent grade. Thiamine hydrochloride was obtained from ACROS Organics. Riboflavin, trichloroacetic acid, sodium acetate, and sodium 1-hexanesulfonate were obtained from Alfa Aesar. Methanol and acetonitrile were obtained from EMD Chemicals. Glacial acetic acid and hydrochloric acid were purchased from Fisher scientific. Triethylamine and takadiastase for protein degradation (originated from aspergillus oryzae powder, Lot#BCBV9342) were bought from Sigma Aldrich.‎ The mushrooms were grown at a local farm and several varieties of mushroom were used (Meridzo Agribusiness Center, Lynch, Kentucky, USA) and harvested, dried, and then ground into powder with a small food processor. The powder was a blend of six natural strains of Shitaki mushrooms (WR 46, 510, 910, 912, Warm, and Wild).

### 2.2. Extraction Method

**Figure 1:**
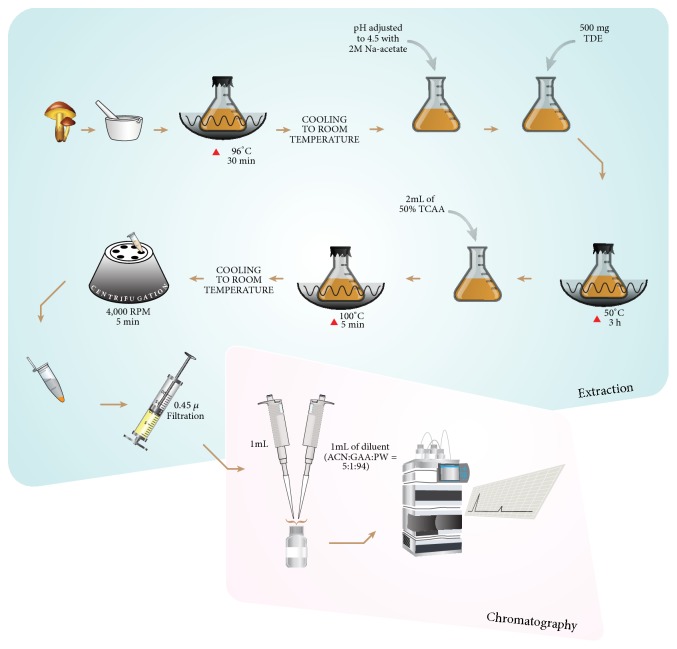
Schematic diagram of the mushrooms extraction method.

The extraction method (Figure 1) used in this study was based on the conditions described by Esteve, Farré et al. 2001 [20, 21]. In brief, the homogenized mushroom powder (2 g) was placed in 40 mL of 0.1M HCl solution in a conical flask covered with aluminum foil and heated at 96°C in a water bath for 30 min. Then, the contents were allowed to cool at room temperature. After cooling, the pH value was adjusted to 4.5 with ~2 mL of 2 M sodium acetate solution. Then 500 mg (concentration of enzyme was 11.90 mg/mL) of takadiastase enzyme was added to the preparation and incubated in a water bath for 3 h at 50°C. Finally, 2 mL of 50% (w/v) trichloroacetic acid solution was added to the preparation and was then heated at 100°C for 5 min. The preparation was then allowed to cool to room temperature and centrifuged at 4000rpm for 5 min. The supernatant was filtered through a 0.45*μ*m membrane filter. After that, 1.0 mL of the filtered solution and 1.0 mL of the diluent (mixture of acetonitrile, glacial acetic acid, and purified water in the ratio of 5:1:94) [11] were transferred to an Eppendorf tube and vortexed. One hundred microliters (100 *μ*l) of this solution was injected into the HPLC system to quantify the vitamin B content. All the reaction vessels were covered under aluminum foil in order to prevent photo degradation of the analytes.

### 2.3. Chromatographic Conditions

The chromatographic methods used to determine the content of vitamins were adopted and modified from the USP to achieve a suitable separation of the two vitamins present in mushrooms [11, 13]. The chromatographic separation of the two water soluble vitamins (B1 and B2) was estimated by using the Shimadzu prominence HPLC system, using an isocratic elution mode (mobile phase A/mobile phase B: 80/20) on a Waters Xterra® MS C-18 column (4.6mm × 150mm, 5 *μ*m) column integrated with a XBridge® BEH C18 Guard column (2.1mm × 5 mm, 5 *μ*m) (Figure 5(a)). The HPLC system consisted of a quaternary pump (LC-20AD), a UV-Vis detector (SPD-20AC, having exceptional level of sensitivity and stability). Mobile phase A consisted of 893mL of 8.0 mM of sodium-1-hexanesulfonate, 7.5 mL of glacial acetic acid, 100mL of methanol, and pH adjusted to 3.0 with the ion-pairing reagent “diethylamine” to enhance peak shape. Mobile phase B consisted of HPLC grade methanol. The instrumental set-up included the flow rate at 1.0 mL/min and the temperature of the column oven was at 30°C. The injection volume was set to 100 *μ*L with a run time of 12 minutes. The effluent was detected at two different wavelengths (245nm-B1, 268nm-B2) based on the lambda max (Figures 3 and 4) obtained using photodiode array detector (PDA, 2996-Waters) to get maximum detector response and sensitivity. System suitability requirement: the resolution between B2 and B1 should be more than 3.5 in the reference standards chromatogram.

Standard stock solutions for thiamine and riboflavin were prepared as per the method outlined in the USP [11]. In brief 20 mg of thiamine hydrochloride and 10 mg of riboflavin were transferred to a 100-mL volumetric flask, and 90 mL of diluent was then added. After combining the components, they were heated in a water bath at 65°C for 5 min with regular agitation. The contents were kept in a cold-water bath for 10 min to cool to room temperature, and the volume was made up to 100 mL using diluent as described earlier (mixture of acetonitrile, glacial acetic acid, and purified water in the ratio of 5:1:94). Finally, 5 *μ*g/mL of thiamine hydrochloride and 2.5 *μ*g/mL of riboflavin mixture solution were prepared in diluting solvent to quantify the respective vitamins present in the test solution.

## 3. Results and Discussion

Unlike commercial or compounded pharmaceutical preparations, phytochemical samples harvested from nature require laborious and sophisticated approaches to purify, extract, and analyze the chemicals. Extraction and quantification are the two key phases of this process. For mushroom samples, the extraction process usually involves hot acid hydrolysis to digest the proteins, with which the vitamins usually form complexes. The acid hydrolysis was followed by enzymatic hydrolysis to cleave phosphate esters and liberate the vitamins. The extraction methodology used in this study was validated, verified, and reported several times [20, 21, 24]. RP-HPLC (UV-Vis Detector) technique is a widely accepted method for identification and quantification of organic compounds in food products and pharmaceutical products. The chromatographic methods (Figure 5(a)) were adopted and modified from the USP to quantify the vitamins B1 and B2 present in mushrooms and were verified by evaluating the specificity, sensitivity, linearity, limit of detection (LOD), limit of quantitation (LOQ), accuracy, reproducibility, robustness, and solution stability [25].

Specificity is the ability to measure the correct and unadulterated analyte in the presence of other components, such as degradants, and the matrix. Specificity is the most important parameter considered during method development, since the products sourced from nature are abundant in myriad types of compounds. The specificity for the chromatographic method used in this study was confirmed by checking the blank interference, resolution between the adjacent peaks, and peak purity (spectral analysis) [26]. Figures 3 and 4 represent the HPLC chromatograms for identification and purity plots with spectral analysis data of B2 and B1 obtained from standards and sample with reference standards, respectively, using Waters photodiode array detector. The spectral analysis was achieved using vector analysis algorithms to evaluate the peak purity and the peak is considered spectrally homogeneous when the purity angle is lower than the purity threshold [26]. In our experimentation, the purity angles have been found to be lower than the purity thresholds for B2 and B1 peaks, in both samples spiked with reference standards and reference solutions. This indubitably confirms that the peaks are spectrally homogeneous and contain only one compound. The retention time for B2 and B1 is ~6.6 min and ~9.2 min, respectively. All of the peaks are symmetrical (Tailing <1.2) in shape and well separated from each other. The resolution between B2 and B1 is more than 3.5 in the reference standards chromatogram.  Figure 5(b) represents the HPLC chromatogram of the diluting solution, which clearly indicates that the diluent and mobile phase have no interference in the studies because no peak is coeluting at the retention time for B2 and B1 peaks. Both of the spiked chromatograms (Figures 3–5) visibly indicate that peaks are well separated from each other, and the resolution between the neighboring peaks is more than 1.5 [18].

**Figure 2:**
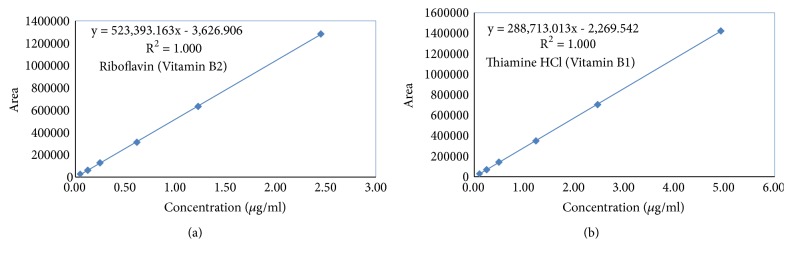
Linear curves for (a) riboflavin (0.05 *μ*g /mL to 2.45 *μ*g/mL) and (b) thiamine hydrochloride (0.10 *μ*g/mL to 4.93 *μ*g /mL).

**Figure 3:**
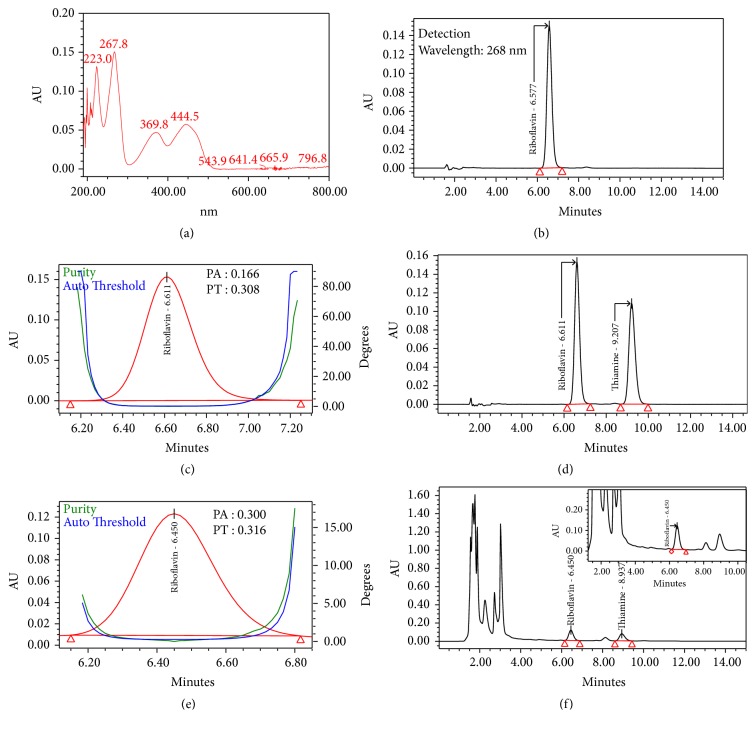
HPLC chromatograms obtained in peak identification and spectral analysis of riboflavin (B2) using photodiode array detector (PDA, 2996-Waters): (a) absorbance spectra of B2 from 190nm to 800nm, (b) reference standard, (c) purity plot of the B2 in standard solution, (d) reference standards solution, (e) purity plot of the B2 in test sample spiked with reference standards, and (f) test sample spiked with reference standards.

**Figure 4:**
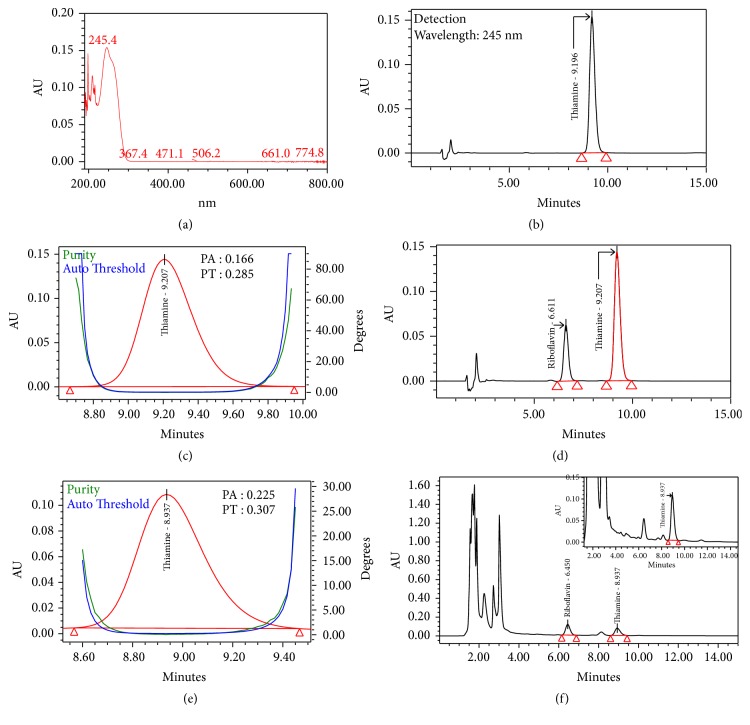
HPLC chromatograms obtained in peak identification and spectral analysis of thiamine (B1) using photodiode array detector (PDA, 2996-Waters): (a) absorbance spectra of B2 from 190nm to 800nm, (b) reference standard, (c) purity plot of the B1 in standard solution, (d) reference standards solution, (e) purity plot of the B1 in test sample spiked with reference standards, and (f) test sample spiked with reference standards.

**Figure 5:**
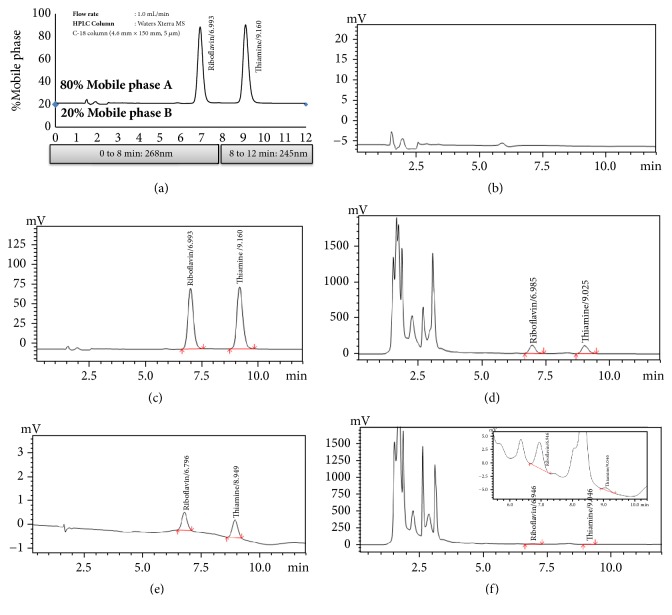
HPLC chromatograms obtained from Shimadzu prominence HPLC system with a UV-Vis detector (SPD-20AC) in method validation studies: (a) isocratic elution parameters, (b) diluting solution, (c) reference standards solution, (d) test sample spiked with reference standards, (e) LOD solution, and (f) test sample (Shitaki mushrooms).

The responses for the proposed method were found to be linear in the following ranges: 0.05 *μ*g/mL to 2.45 *μ*g/mL (y = 523393x-3627, R^2^ = 1.00) and 0.1 *μ*g /mL to 4.93 *μ*g/mL (y = 288713x-2270, R^2^ = 1.00) for B2 and B1, respectively (Figure 2). LOD's and LOQ's values were calculated based on the standard deviation of the response (*σ*) and the slope of the calibration curve (S) according to the formula: LOD = 3.3*σ* /S, LOQ= 10*σ* /S as per ICH guidelines [27]. LOD for B2 and B1 were 0.029 and 0.043 *μ*g/mL, and LOQ for B2 and B1 were 0.088 and 0.132 *μ*g/mL, respectively. The highest responses were obtained because the effluent was detected at two different wavelengths (245nm-B1 and 268nm-B2) based on their lambda max (Figures 3 and 4). The standard and sample solutions for B2 and B1 were found to be stable at 72 hours at controlled cold temperature/refrigerator for 72 h. The accuracy and precision of the method were determined by spiking known concentration of these two vitamins in the 2.0 g blend of six natural strains of Shitaki mushrooms (WR 46, 510, 910, 912, Warm, and Wild). Recovery studies were carried out with three replicates in two different days. The % recovery for B2 and B1 was found to be within 90% -110% with a % RSD less than 5.0. The % recovery (range), mean, and standard deviation (SD) were reported in Table 1.

The chromatographic conditions, especially selecting the most suitable mobile phase, are the most important parameter for HPLC method. Low concentrations of salt help increase column longevity and reduce column backpressure. The mobile preparation procedure for this proposed method is very simple and contains very low concentration of salt (8.0 mM of sodium-1-hexanesulfonate). The mobile phase was also prepared in two different days and showed reproducible results; the pH of the mobile phase was adjusted to 3.0 with diethylamine. Replicate injections of a standard preparation were used to ascertain the system precision. The percentage relative standard deviation of the three replicates of the standard solutions was found to be less than 1.0, indicating that the HPLC system was very precise during analysis. The specificity and suitability of the method were verified using two different HPLC columns: Waters X-Select CSH C-18 column (4.6 mm × 150 mm, 5.0 *μ*m particle size,) and Waters Xterra® MS C-18 column (4.6mm × 150mm, 5 *μ*m) integrated with a XBridge® BEH C18 Guard column (2.1mm × 5 mm, 5 *μ*m) and the resolution between adjacent peaks was evaluated. Both columns are found to be suitable for the method, but Xterra is found to be more suitable than X-Select in view of resolution between B1 and B2. The robustness of the method was evaluated by changing the optimized condition of the mobile phase ratio (percentage of mobile phases A and B ± 10%) and the resolution between adjacent peaks was evaluated. Based on the experimental data obtained thereby, we can conclude that the method is robust enough.

The extraction involved acid hydrolysis followed by enzymatic dephosphorylation with takadiastase enzyme. Some researchers in the past have chosen to omit the enzymatic dephosphorylation step and tried or attempted to continue without cleaving the phosphate ester bonds [20]. In this study, these two steps were monitored by HPLC to evaluate the importance of the enzymatic dephosphorylation step in mushroom analysis. The dephosphorylation step is very important for mushrooms analysis because there are significant differences between the two steps (more than 15% difference between the results obtained from the two steps). The content obtained for B1 and B2 in mushrooms after enzymatic dephosphorylation step has been reported in Table 1.

## 4. Conclusion

The obtained experimental data collectively confirm that the method we developed for the quantitative and qualitative analysis of mushroom products was simple, specific, linear, accurate, reproducible, and sensitive enough to detect and quantify these two water soluble vitamins in mushroom samples. This method may be applied for rapid analysis and quantitation of thiamine and riboflavin in mushroom contents, in food industry during routine analysis.

## Figures and Tables

**Table 1:** Method validation results.

Validation Parameters	Riboflavin (B2)	Thiamine (B1)
Detection Wavelength	268 nm	245 nm
Retention Time	~ 6.0-7.0 min	~ 9.0 -9.5 min
Peak Purity-Standard	PA: 0.166 < PT: 0.308	PA: 0.166 < PT: 0.285
Peak Purity-Spiked	PA: 0.300 < PT: 0.316	PA: 0.225 < PT: 0.307
Linearity	y = 523393x-3627, R^2^ = 1.00	y = 288713x-2270, R^2^ = 1.00
Limit of detection (LOD)	0.029*μ*g/mL	0.043*μ*g/mL
Limit of quantitation (LOQ)	0.088*μ*g/mL	0.132*μ*g/mL
%Recovery & Precision (Mean ±SD)	104.88±2.04 (103% -107%)	104.45±4.5 (101% – 109%)
System Precision (%RSD of Area)	0.63	1.17
%Recovery & Intermediate Precision	103.35±2.80 (101% – 106%)	101.97±2.43 (99% -104%)
Solution stability (Standard & Sample)	72 hours at controlled cold temperature/Refrigerator	

Robustness data	Resolution between Riboflavin and Thiamine	

HPLC Column 1 (Xterra MS C-18)	4.6 (Retention Time: 7.0/9.2)	
HPLC Column 2 (X-Select C-18)	3.5 (Retention Time: 7.0/8.5)	
Mobile Phase Ratio (78:22)	4.3 (Retention Time: 6.9/9.0)	
Mobile Phase Ratio (80:20)	4.6 (Retention Time: 7.0/9.2)	
Mobile Phase Ratio (82:18)	4.6 (Retention Time: 8.2/9.9)	

Sample Analysis (Mean in *μ*g/g ±SD) (Shitaki mushrooms)	Day 1: 5.95±0.44	Day 1: 0.93±0.07
	Day 2: 6.03±0.27	Day 2: 1.17±0.001

PA: purity angle and PT: purity threshold, blend (six-month-old) of six natural strains of Shitaki mushrooms.

## Data Availability

All the data used to support the findings of this study are stated and included in the article.
